# Editorial: Targeting Indoleamine 2,3-dioxygenases and Tryptophan Dioxygenase for Cancer Immunotherapy

**DOI:** 10.3389/fimmu.2021.789473

**Published:** 2021-12-06

**Authors:** Lieve Brochez, Vibeke Kruse, Dirk Schadendorf, Alexander J. Muller, George C. Prendergast

**Affiliations:** ^1^ Department of Dermatology, University Hospital Ghent and Cancer Research Institute Ghent (CRIG), Ghent, Belgium; ^2^ Department of Medical Oncology, University Hospital Ghent and Cancer Research Institute Ghent (CRIG), Ghent, Belgium; ^3^ Department of Dermatology and Comprehensive Cancer Center, University Hospital Essen, Essen, Germany; ^4^ Lankenau Institute for Medical Research, Wynnewood, PA, United States

**Keywords:** IDO, TDO, cancer immunology, immunotherapy, immunometabolism

Immunometabolism is emerging as a core element in cancer, both in the pathophysiological equation that yields malignancy and in the therapeutic equation that yields curative efficacy. In particular, the tryptophan catabolic enzyme IDO1 has attracted much attention as a functional biomarker and therapeutic target in many cancers, with increasing notice of TDO, IDO2 (IDO/TDO enzymes), along with TPH and IL4I1 as additional tryptophan-degrading enzymes that drive malignancy and impede therapy *via* enzymatic and non-enzymatic processes like IDO1. A rapidly growing literature reveals multiple mechanisms through which these bad actors subvert tumor microenvironments to drive disease, most notably by tolerizing the immune system to tumor antigens, but also by enhancing tumor-feeding inflammatory signals and abnormal vascular networks. While initially focused on IDO1, which multiple studies have correlated with negative clinical outcomes (1), the full scope of drug discovery and development efforts now encompasses all these tryptophan-degrading enzymes, reinforced in part in 2018 by the negative readout of a large but flawed Phase 3 trial of the IDO1-selective inhibitor epacadostat in combination with the PD1 antibody pembrolizumab in stage IV melanoma (2). It is clear further insights into the expression and function of IDO1 and other tryptophan-degrading enzymes is needed in cancer. 

This Research Topic includes two reviews on IDO/TDO enzymes in cancer. Zhai et al. survey IDO1-mediated immunosuppression in advanced brain tumors (glioblastomas), where IDO1 elevation synergizes with naturally occurring elevation of IDO1 in the central nervous system during aging. Notably, findings presented argue that immunotherapeutic efficacy in this setting requires neutralization of both the enzyme-dependent and enzyme-independent functions of IDO1. While enzyme inhibitors lack dual-targeting capability, vaccine approaches to eliminate IDO1 protein may be useful in this challenging disease setting (3, 4). Meireson et al. reviewed induction mechanisms and distinct functions of IDO1 in different compartments of the tumor microenvironment, including tumor cells, tumor stromal fibroblasts, endothelial, immune and mesenchymal cells, and peripheral blood. This group has recently published data on the clinical importance of IDO1 expression and its IFNγ-induced upregulation in peripheral blood monocytes in early stage melanoma (5).

Genetic experiments in mice support the likelihood that IDO1 functions in both tumor cells and stromal immune cells to drive immunosuppression (6). A recent extension of these genetic studies suggests that IDO1 may drive metastatic growth by enabling tumor neovascularization, through an innate inflammatory pathway distinct from adaptive immune control (7, 8). The complexity revealed in diverse patterns of IDO1 expression in human tumors may impact how IDO1 blockade influences therapeutic responses in different drug combination contexts.

This Research Topic also includes six research reports on IDO/TDO enzymes in cancer, four of which address the potential prognostic utility of IDO/TDO expression in various disease settings. Herrera-Rios et al. studied IDO1 expression in brain metastases of human melanoma. As noted by Zhai et al., the immunosuppressive properties of brain parenchyma differ from the rest of the body. Here a comparative tissue analysis identified macrophages/microglia as the major source of IDO1 expression, which was observed to correlate with a specific reduction in cytotoxic CD8+ T cells. Thüring et al. conducted a pilot clinical study of IDO1 gene expression in urine obtained from prostate cancer patients undergoing radical prostatectomy, where they observed evidence of a correlation with Gleason progression score. In human hepatocellular carcinoma, Chinnadurai et al. implicated IDO1 and PD-L1 as players in T cell suppression in this setting by investigating RNA expression of IDO/TDO and a set of B7 ligands. Lastly, in a retrospective study of non-small cell lung cancer (NSCLC), Mandarano et al. report a correlation between elevated levels of IDO2 and poor prognosis, a finding recently confirmed by another group (9).

Two studies in this Research Topic probed the relationship between IDO/TDO enzymes and therapeutic response. In the Lewis model of lung cancer, Zhang et al. combined a targeted IDO1 siRNA with photothermal therapy (PTT), an irradiative modality that may engage an abscopal mechanism of antitumor immunity. Using a nanocarrier approach to coordinately deliver the siRNA and enable tumor heating, the authors observed that IDO1 downregulation stimulated antitumor immunity in combination with PTT (which appears to act by stimulating tumor cell apoptosis). These findings extend the evidence that the efficacy of specific anti-cancer regimens such as PTT can be improved by IDO1 blockade (1, 10). Botticelli et al. studied patients with NSCLC, renal cell carcinoma (RCC) or head and neck squamous carcinoma (HNSCC) who were treated with anti-PD1 after failing first-line therapy. Their findings suggested a correlation between elevated serum Kyn/Trp ratio and early progressors in squamous and non-squamous NSCLC. Further, they observed a trend toward longer progression-free survival when the serum Kyn/Trp ratio was determined to be at normal baseline levels. These observations align with other findings where baseline Kyn/Trp levels, but also increased levels at week 4 of anti-PD1 immunotherapy, were associated with decreased survival in melanoma and renal cell carcinoma (11). In the study from Botticelli et al., while some correlation to gender, site of metastasis, NSCLC and squamous histology was observed, the suggestive data obtained will require follow up in larger studies. Contextually focused studies such as these are critical to informing how treatments targeting IDO/TDO enzymes can be best leveraged for future clinical success.

## Author Contributions

GP and LB composed the text. All authors contributed to the article and approved the submitted version.

## Funding

GP acknowledges generous support for IDO/TDO studies over nearly two decades by NIH grants R01 CA109542, R01 CA191191 and R21 CA 159337 and support from the U.S. Department of Defense Breast Cancer Research Program, American Lung Association, Dan and Florence Green Foundation, Charlotte Geyer Foundation, Pardee Foundation, W.W. Smith Trust, New Link Genetics Corporation, Lankenau Medical Center Foundation and Main Line Health. This funding was not related to the writing of this Editorial. This research program has benefited from long-standing productive collaborations with Alexander Muller, James DuHadaway, Lisa Laury-Kleintop and Laura Mandik-Nayak and many others at Lankenau. LB: Part of this research was supported by ‘Kom Op tegen Kanker’ (Stand up against Cancer).

## Conflict of Interest

LB has participated in an advisory board to Incyte Inc. on epacadostat in melanoma and was invited to give a MSL seminar to Incyte Inc. on IDO (2017). GP declares a conflict of interest due to his role as a co-inventor in the discovery and development of IDO/TDO inhibitor technologies patented by the Lankenau Institute for Medical Research and licensed to Duet Therapeutics, Inc., a private company for which he serves presently as a scientific advisor.

The remaining authors declare that the research was conducted in the absence of any commercial or financial relationships that could be construed as a potential conflict of interest.

## Publisher’s Note

All claims expressed in this article are solely those of the authors and do not necessarily represent those of their affiliated organizations, or those of the publisher, the editors and the reviewers. Any product that may be evaluated in this article, or claim that may be made by its manufacturer, is not guaranteed or endorsed by the publisher.
